# Radiographic and histologic assessment of osseointegration for surface-treated titanium dental implants: An experimental study in dogs

**DOI:** 10.34172/joddd.41009

**Published:** 2024-03-29

**Authors:** Mohammed A Abdulla, Radhwan H Hasan, Osama Hazim Al-Hyani

**Affiliations:** ^1^Department of Prosthetic Dentistry, College of Dentistry, University of Mosul, Mosul, Iraq; ^2^Department of Surgery and Theriogenology, College of Veterinary Medicine, University of Mosul, Mosul, Iraq

**Keywords:** Delayed Loading, Osseointegration, Surface treatments, Titanium implants

## Abstract

**Background.:**

Modifying the dental implant’s surface texture enhances its biological response to the bone and implant‒tissue interface, resulting in the successful support of forces. This study assessed the impact of sandblasting, sandblasting plus acid etching, Er,Cr:YSGG laser, and propolis implant surface treatments and occlusal load on the osseointegration of titanium dental implants in dogs.

**Methods.:**

Seventy-two titanium dental implants with a diameter of 4 mm and a length of 10 mm were divided into four groups according to implant surface modifications (n=18 for each group): group A: sandblasting plus acid etching, group B: sandblasting with Al_2_ O_3_, group C: Er,Cr:YSGG laser, group D: propolis coating. Twenty-four local breed male dogs were used. Premolar teeth (P1, P2, and P3) were extracted on the left side of the mandible, and after 12 weeks of bone healing, implants were unilaterally installed. The osseointegration at three study times from implant installation (14, 90, and 180 days) was evaluated. The dog jaws were scanned using an intraoral scanner for the virtual design of screw-retained three-unit crowns after 90 days of osseointegration. Final radiographs were taken before the animals were sacrificed at 14, 90, and 180 days, and the histological analysis was performed.

**Results.:**

Radiographic analysis showed new bone formation (NBF) along and in contact with the implant surface of the treated groups. The histological analysis after 14 days in groups A and B revealed a uniform and ongoing pattern of bone growth and many osteoblasts with few osteocytes within lacunae in new bone trabeculae. Group C showed an increase in the number of osteoblasts lining thin bone trabeculae. Group D showed a generative power concerning bone. At 90 days, there was increased bone ingrowth, and the new bone matured in all the treated implant groups. At 180 days, dense mature bone apposition was in direct contact with delayed-loaded implant surfaces.

**Conclusion.:**

A radiographic examination revealed that surface modification significantly impacted osseointegration, with a strong bond between the implant surface and the surrounding bone. The histological sections at the 14-day interval revealed obvious bone remodeling activity, especially in sandblasting plus acid etching and sandblasting-modified implant surface groups. At the 90-day interval, bone ingrowth had increased, and the new bone became mature, especially in sandblasting and propolis surface modification groups. After 180 days of the delayed-loaded implant osseointegration, differences were observed between different implant-treated groups with a remarkable remodeling of the bone, especially in the propolis coating group.

## Introduction

 Titanium and its alloys are suitable for biomedical applications due to their high specific strength and biocompatibility.^1^ The selection of the biomaterial is now situational, based on the characteristics of the local tissue.^2^

 Osseointegration has a significant role in dental implant success. For optimal osseointegration to occur without interfering with the connective tissue layer, there must be direct contact and interaction between the peri-implant tissues and implant surfaces.^3,4^

 Numerous surface modification techniques have been developed to improve the compatibility of titanium and the osseointegration of surrounding bone structures. These techniques include laser surface modification, anodization, hydroxyapatite coating, sandblasting, acid etching, or a combination of the two, and Ti plasma spray coating. A rough surface can increase bone-to-implant contact; changes in the implant surface’s roughness also significantly affect how well the surrounding bone heals.^5-7^ Concerns about late implant failure brought on by the loss of osseointegration after functional loading have persisted for the last decade.^8^

 According to conventional protocols, implants should not be loaded throughout the osseointegration phase, typically lasting 3‒4 months for the mandible and 6‒8 months for the maxilla.^9,10^ Bone healing increases the contact between the implant and the surrounding bone after insertion. An implant may fail if loaded before this contact reaches a particular point. Because of this, the implant stability outcome should be used to determine the loading time. Optimal occlusal forces may result in adaptive bone remodeling, improved osseointegration, and greater bone-to-implant connections.^11^ This creates a permanent bond between the implant under load and the surrounding bone, with the direction and intensity of the applied loads, the remodeling and bone degradation processes determined.^12^

 The current study assessed the impact of sandblasting, sandblasting plus acid etching, Er,Cr:YSGG laser, and propolis coating of the implant surface radiographically and histologically with and without occlusal loads on the osseointegration of titanium dental implants in dog models.

## Methods

 This experimental animal study was conducted at the experimental surgical center of the Veterinary Teaching Hospital, the College of Veterinary Medicine, University of Mosul.

###  Study setting

 Twenty-fourhealthy mature adult local breed male dogs were included in the current study, aged 1‒1.5 years and weighing (22 ± 3 kg). Throughout the study, the animals were provided with standard laboratory nutritional support, proper veterinarian treatment, and care according to institution guidelines. Throughout the experimental study, each healthy dog was housed individually in a 1.5-m^2^ cage under a 12-hour light/dark cycle and fed with natural food with water available. Each dog was subjected to oral hygiene and plaque control by regular mechanical cleaning of both teeth and implants using a mechanical toothbrush and 0.2% chlorhexidine mouth irrigation once a week during the study period by the veterinary assistant who was familiar with experimental dogs under the supervision of the veterinarian. The assessment of the oral hygiene condition was based on a protocol that assessed plaque and calculus, according to Bellows.^13^ Sometimes, an ultrasonic scaler was used in certain cases under anesthesia.

###  Study design

 According to a previous study by Abdulla et al,^14^ the titanium dental implant fixture investigated in this study was Dentium®, a standard screw-type dental implant system (Dentium Co., Ltd. Seoul, Korea), with a diameter of 4 mm and a length of 10 mm. A total of 72 commercial, pure titanium dental implants were randomly divided into four groups according to the surface treatments:

- Group A: (n = 18) titanium dental implants; sandblasting plus acid etching (SLActive) surface treatment (etched with warm hydrochloric acid [HCl] concentration of 37% at 60ºC for 5 minutes, rinsed and cleaned by the ultrasonication method in ultra-pure water, and dried).
- Group B: (n = 18) titanium dental implants; sandblast surface treatment (air-abraded with 50-μm aluminum oxide [Al_2_O_3_] particles for 15 s at 0.6 MPa, 6 bars of pressure).
- Group C: (n = 18) titanium dental implants; Er,Cr:YSGG laser surface treatment (at a wavelength of 2780 nm, set at 100 mJ/pulse, with a power of 2.5 W, a frequency of 30 Hz, and pulse duration of 60 seconds, accompanied by 40% water and 50% air spray).
- Group D: (n = 18) titanium dental implants; propolis coating surface treatment (the aqueous ethanolic extract of propolis [100 mg/mL] had a pH of 6.2, was applied in drops, brushed on the demarcated areas of the implant surface with a brush tip, and allowed to adhere for 15 s to the titanium implant surface).
- Twenty-four local breed male dogs, 1‒1.5 years and weighing 22 ± 3 kg, were randomly divided into three groups (n = 8 for each group) according to the time of sacrifice as follows:
- Group I: sacrifice at 14 days from implant installation (unloaded)
- Group II: sacrifice at 90 days from implant installation (unloaded)
- Group III: sacrifice at 180 days from implant installation (loaded)

###  Surgical procedure 

 Before each surgical procedure, the dog was starved for 12 hours, the mouth was rinsed with 0.2% chlorhexidine mouthwash, and systemic prophylactic antibiotics were administered by a combination of procaine penicillin and streptomycin (IM) at a dose of 10 000 IU per 10 mg/kg weight. Analgesia was achieved with Metalgen at a dose of 3 mL once daily and continued for four days after the operation.

 The chosen animal underwent general anesthesia on the day of the procedure after being examined by a veterinarian, using an intramuscular injection of 10% ketamine hydrochloride (10 mg/kg body weight) with 20% xylazine at a dose of 2 mg/kg intramuscularly, which kept the animal sedated for the required time with minimal pain. The animals were pre-anesthetized, and a conventional dental infiltration local anesthetic agent, 2% lidocaine HCL with epinephrine 1:80 000, was administered through injection into the buccal and lingual gingiva at the surgical site for hemostasis.

###  Extraction phase

**Figure 1:**
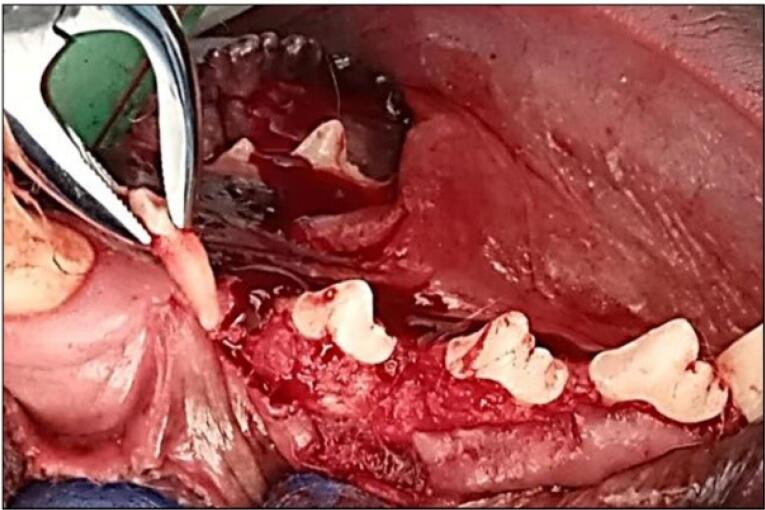
Surgical extraction of mandibular P1, p2, P3

 The mandibular left premolar teeth (P1, P2, and P3) were extracted (Figure 1). A supracrestal incision was made from the mandibular canine to the first molar (M1). A mucoperiosteal full-thickness flap both buccally and lingually elevated, and using a tungsten carbide bur, the teeth were sectioned buccolingually at the bifurcation. Then, the roots were extracted one at a time using elevators to remove any separated root remnants, and lower surgical forceps were not used so as not to damage the remaining socket bone walls. The dimensions of the sockets were measured using digital calipers, and mean alveolar ridge measurements were determined. The flaps were repositioned using multiple sutures for a 12-week healing period after tooth extraction. The dogs were fed a soft diet, and the sutures were removed after two weeks.

###  Implant placement phase

 After 12 weeks of healing and adequate bone healing (adequate bone formation) in the socket of the extracted left mandibular premolars P1, P2, and P3 teeth, three dental titanium implants (Dentium Co., Korea) (4 × 10 mm in diameter and length, respectively) were installed in the position of the previously extracted mandibular premolars (P1, P2, and P3).

**Figure 2:**
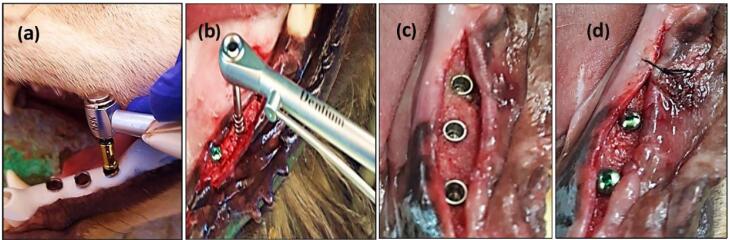
The osteotomy and implant installation A: Customized surgical guide during implantation within dogs’ oral cavity. B: An implant was installed at an insertion torque of ≥ 35 Ncm. C: Cover screwed at 20 Ncm torque. D: Soft tissues were closed with non-resorbable sutures

 The surgical implant placement protocol and the sequential osteotomy were performed according to the manufacturer’s guidelines at the recipient sites using a surgical guide template with the sequential steps of implant placement (Figure 2: a, b, c, and d). The method is thought to minimize remaining bone loss and minimize micro-deformations. First, a pilot drill (diameter: 2.2 mm, depth: 10 mm) was prepared at 1000 rpm (30‒45 Ncm) with irrigation of sterile 0.9% saline solution. The cavity was then gradually made wider with a secondary drill that had a larger diameter. 1000 rpm (30‒45 Ncm) with irrigation was used until the final diameter for the implant was achieved, and a condensing drill was used at 50 rpm (30‒45 Ncm) with irrigation (a diameter of 4 mm and a depth of 10 mm). After preparation, the holes were cleaned, and the implants were installed at a rotation of 50 rpm (30‒45 Ncm) without irrigation. The surgical torque control (insertion torque) was ≥ 35 Ncm. Subsequently, cover screws (Dentium Co., Ltd. 150, Eondong-ro, Giheung-gu, 16985, Republic of Korea) were screwed at 10 Ncm on each implant to allow a submerged healing protocol, and the soft tissues were closed with non-resorbable sutures. The flap was repositioned and sutured using multiple sutures over the cover screw. The dogs were fed a soft meal, and the local wound area was cleaned with 0.12% chlorhexidine. The sutures were removed after two weeks. The osseointegration at three study time intervals after implant installation was evaluated. Group I dogs were sacrificed after 14 days (implant fixture without loading), and group II dogs were sacrificed after 90 days (implant fixture without loading).

**Figure 3:**
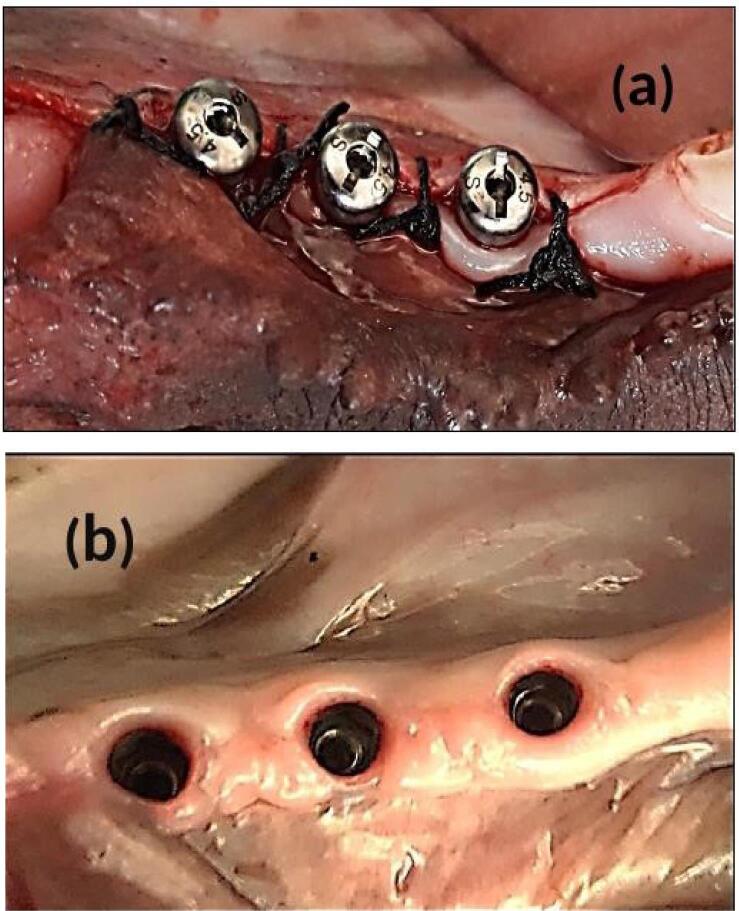
After 12 weeks of implant installation. A:Standard 3.5 mm height healing abutments were screwed. B: After 14 days of healing, the gingival tissue cuffs ( Gingival collar)

 For group III dogs (implant fixture with delayed loading), the cover screws were removed and replaced by standard (4.5 mm in diameter, 2 G/H, 3.5 mm in height) healing abutments (Dentium Co., Ltd. 150, Eondong-ro, Giheung-gu, 16985, Republic of Korea). After 14 days of gingival healing, the sutures were removed. The reshaping of the peri-implant healthy attached gingival pink tissue cuffs (gingival collar) was observed, and the healing abutments were removed (Figure 3, a and b).

###  Scanning technique and implant-supported prosthesis

**Figure 4:**
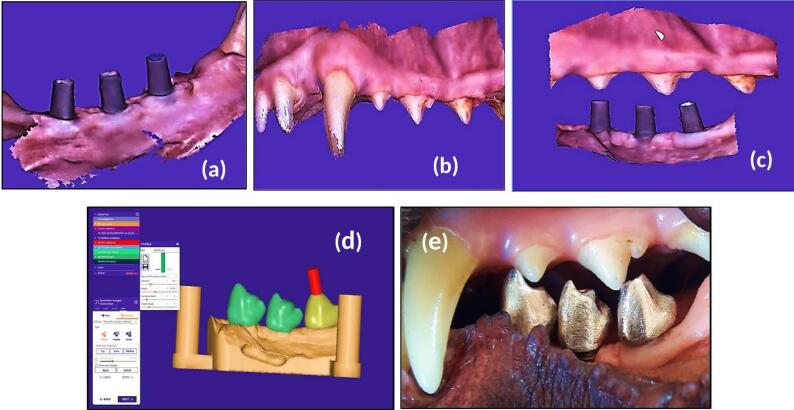
After 14 days of suture removal. A: A working scanned model. B: An antagonist scanned arch. C: Bite registration in the maximum intercuspal position (MIP). D: The virtual design of the 3D printed model. E: Screw-retained full-metal crown intraorally

 For group III dogs, the dog jaws were scanned using an intraoral scanner (3DISC Heron^TM^, HERON SCAN 3.1, IOS Intraoral Color Impression Scanner, USA). This scanning method captures the lingual and buccal surface when scanning the occlusal arc, scanning the areas of interest of each surface. A working jaw with an antagonist arch and bite registration was scanned digitally (Figure 4, a, b, and c) and recorded in the maximum intercuspal position (MIP) at the existing occlusal vertical dimension (OVD). Digital impression was immediately transported to the dental technician’s laboratory, where the splinted full-metal crowns were fabricated.

 Laboratory stages included the digitally scanned models STL file, which was exported to the rapid dental 3D prototyping printer (HALOT-SKY Resin 3D Printer, China) to obtain the 3D-printed model to be used for the construction of screw-retained implant-supported splinted crowns from nickel-titanium metal alloy substructure (RITON titanium alloy powder, China). Data were transferred to the Exocad software (Version 3.1, Germany) for the virtual design of screw-retained full-contour splinted unit crowns (Figure 4d) with the recommended 1-mm occlusal contact between the constructed new crowns and the natural antagonists.

 The fixed dental prosthesis was delivered and screwed with the dental abutment onto the fixtures (Figure 4e). At splinted crowns insertion, recommended torque values were applied to the abutment screws (35 Ncm) and prosthetic screws (15 Ncm), and fundamental one-point occlusal contact between the screw-retained metal crowns and the opposing natural premolars were confirmed using articulating paper at a tolerance thickness of 200 µm. When the implants were functionally loaded, the canines and incisors in centric occlusion, centric contacts, and lateral excursions on all crown surfaces were meticulously examined and adjusted as necessary. The mandibular molars acquire a more vestibular position in this lateral position, which enables the buccal wall of the first mandibular molar to be in effective contact with the lingual wall of the maxillary carnassial (4th premolar). In contrast, the premolars show no contact when the dog bites.

###  Radiographic evaluation

 Using an x-ray machine (POX-300 BT, Toshiba, Rotanode^TM^, Japan), digital radiographs were needed for each animal at the time of implant insertion and 14, 90, and 180 days later to confirm implant osseointegration and evaluate post-surgical crestal bone level alterations. A suitable selection of exposure parameters was made by considering the animal model’s thickness and weight.

###  Animal sacrifice

 After a follow-up period of 14, 90, and 180 days, the animals were sacrificed with an overdose of general anesthetic agent and perfusion through the carotid arteries with a fixative solution. The mandible was freed from the attachment surrounding soft and hard tissues. It was removed using a saw, cut in half at the midline of the anterior part of the mandible, and placed in the fixative/preservative solution. The peri-implant bony tissue was cut in 1-cm size around the implant (about 1 × 1 cm) to achieve a cubic biopsy measuring 2 cm in size with the implant in the middle of this distance. The specimen was longitudinally sectioned in the middle with a diamond circular saw (under a continuous stream of sterile normal saline solution). The specimen was kept in a 10% neutral buffered formalin solution (pH = 7.3) until examined histologically.

###  Histological evaluation

 The histologic and histomorphometric analyses were performed in the Department of Oral and Maxillofacial Surgery, Oral Pathology, the College of Dentistry, University of Mosul.

 After fixation, the specimen was decalcified with 5%‒10% formic acid decalcifying solution and then HCI at 5%‒10% concentration. The specimen was dehydrated slowly through graded ethanol alcohol, 70%, 80%, 90%, and 100%, respectively. The specimens were placed on a slide after being molded in the middle of the paraffin block and adjusted to a microtome for serial sectioning at a 4‒5-μm thickness. The slide was submerged in a container containing hematoxylin and eosin stain to stain the tissue for 10 minutes.

 The slides were subjected to histopathologic and histometric analyses conducted under a light microscope (OPTIKA B-383PL Trinocular Microscope, N-Plan Objectives, Italy). All analyses were performed by an examiner unaware of which group (experimental or control) each sample belonged to. The microscopic finding includes the evaluation of cell-forming bone and bone lamellae. Cell counting and measuring of lamellar thickness was illustrated using a special graduated microscopic lens at a magnification of × 10.

###  Criteria of measurements

 Calibration was done using a microscope-calibrated lens and software application (OPTIKA Proview Program, Italy). A microphotograph of the calibrated slide was taken at the predetermined magnification for measuring:

1. Four randomly selected locations of each slide section were examined.
2. Each location was divided into four quarters by a graduated lens. Image at × 10 magnification was opened using the program application; then, the free hand selection tool was used to outline the required area, followed by selecting a measure for analysis.
3. The measurements were applied to each quarter separately, and the mean was considered for these four measurements of the same slide.
4. The mean of each slide section was used for the biostatistical analysis.

###  Statistical analysis

 The data was analyzed using SPSS 21.0 (Chicago, IL, USA). The statistical data and the descriptive statistics were assessed. One-way ANOVA was used to assess all implant surface modification data, with *P* values of ˂0.05 considered statistically significant. Post hoc Tukey tests were used to compare the significant groups.

## Results

**Figure 5:**
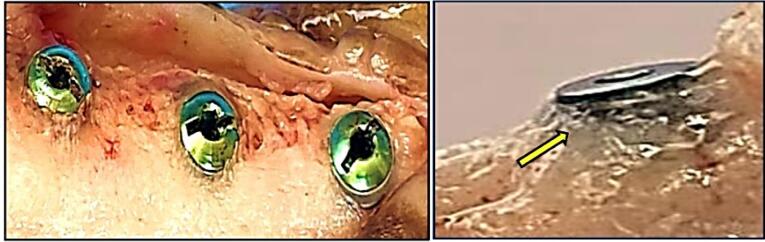
New bone formed around the head of the implant in the treated implant groups (yellow arrow)

 After 14, 90, and 180 days of bone healing, implant surfaces in the present study showed a good bone response with sufficient new bone development along the implant surface. The results confirmed the importance of the modified implant surface regarding the physicochemical properties of dental implants in their osseointegration. Grossly, the coronal head of the implant surface modifications was overhanging the head of the implant in the treated implant groups by newly formed bone tissue (Figure 5). After implant installation, four implants were considered unsuccessful and lost completely after 14, 30, and 90 days.

**Figure 6:**
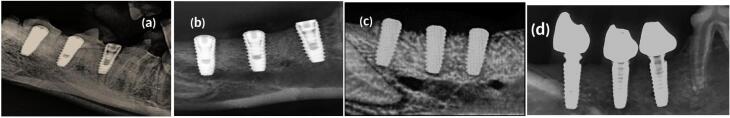
Radiographical evaluation of bone osseointegration; x-ray images were taken (a)at the time of implant installation; (b) after 14 days of impalnt installation; (c) ater 90 days of osseointegration. (d) after 180 days of osseointegration

 A radiographic examination showed no evidence of osseointegration disorders or radiolucent areas. An x-ray scan demonstrated that 68 implants had a smooth osseointegration process. According to the radiographic examination, new bone tissue developed around the mandibular cortical bone at various intervals (Figure 6, a-d). Moreover, the process of bone regeneration persisted to the apical area around the dental implant. This demonstrated that surface roughness and modification significantly impacted osseointegration in dental implants with similar threaded designs. These factors also had excellent biocompatibility and bone-forming capacity, which ensured the mechanical stability of the dental implant.

**Figure 7:**
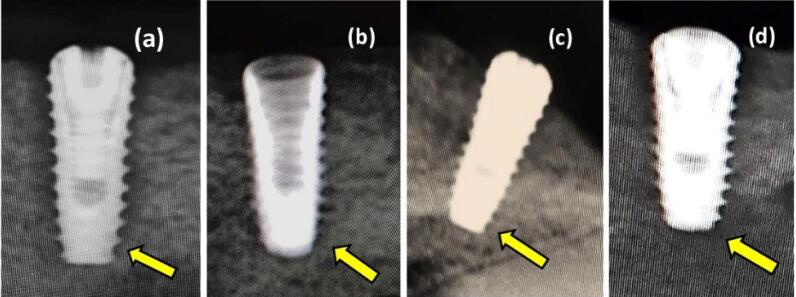
Digital radiographs after 90 days of implant osseointegration. (a) Sandblast with acid etching surface treatment. (b) Sandblast surface treatment. (c) Laser surface treatment. (d) Propolis surface treatment

 A strong bond between the implant surface and the surrounding bone was observed in the apical portion of the implant (Figure 7, a-d). There was a tiny space between the implant and the surface of the bone at the crestal side of the implant. Fibrous tissue filled in this space. The original drill edges were still occasionally recognized.

 Histologically, the cross-section throughout the healing process following implantation revealed minimal granulation tissue and well-organized bone growth. The titanium implant body underwent a sequence of healing events at varying time intervals of sacrifice (14, 90, and 180 days), and the implant space was encircled by newly formed bone.

**Figure 8:**
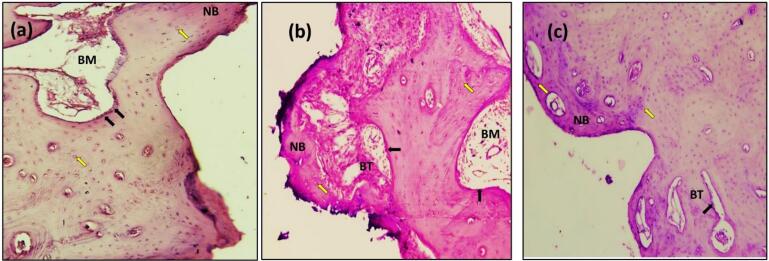
Microscopic photograph view of a cross-section of the sandblast with acid etching implant treatment: (a) 14 days; (b) 90 days; (c) 180 days. (NB): new bone, (BT): bone trabeculae, (BM): bone marrow. Black arrow: Osteoblasts (Ob); Yellow arrow: Osteocytes (Oc); (Hematoxylin-Eosin, magnification = 10X)

**Figure 9:**
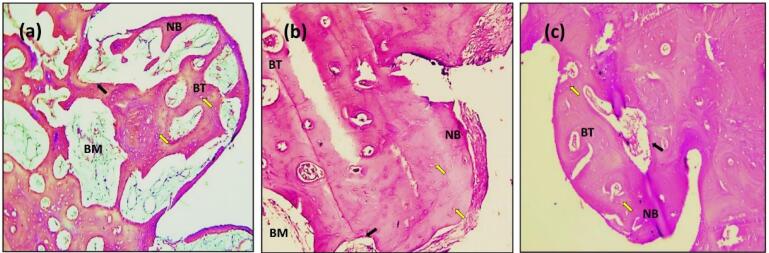
Microscopic photograph view of a cross-section of the sandblasting implant treatment: (a) 14 days; (b) 90 days; (c) 180 days. (NB): new bone, (BT): bone trabeculae, (BM): bone marrow. Black arrow: Osteoblasts (Ob); Yellow arrow: Osteocytes (Oc); (Hematoxylin-Eosin, magnification = 10X)

 In the present study, after 14 days, the sections with H&E staining analysis of the histological sections showed obvious bone remodeling activities. New bone formation was present in all the implant groups with a thin bone trabeculae formation (BT). Although bone formation was present in all the implant groups, sandblasting plus acid etching (Figure 8) and the sandblasting (Figure 9) of the implant surface showed a more uniform and continuous pattern than the other implant groups. The magnifying views at × 10 showed obvious and rapid increases in the number of osteoblasts with the appearance of osteocytes within lacunae in new bone trabeculae. A noticeable seam of osteoblasts and osteoid revealed the ongoing bone production, and a distinct band of immature newly woven bone was seen around the implant surface, extending from the preexisting bone.

**Figure 10:**
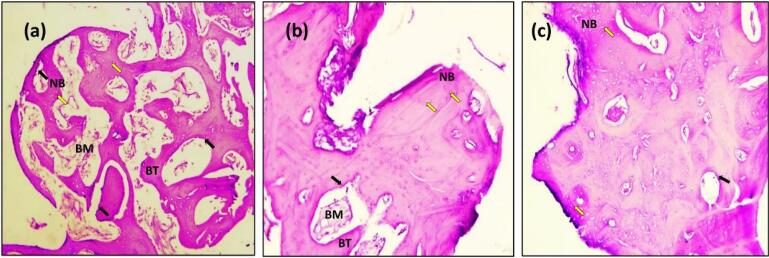
Microscopic photograph view of a cross-section of the Er,Cr:YSGG laser implant treatment: (a) 14 days; (b) 90 days; (c) 180 days. (NB): new bone, (BT): bone trabeculae, (BM): bone marrow. Black arrow: Osteoblasts (Ob); Yellow arrow: Osteocytes (Oc); (Hematoxylin-Eosin, magnification = 10X)

 The histologic feature of the implants treated with Er,Cr:YSGG laser after 14 days of implantation at × 10 showed a gradual increase in the number of osteoblasts that were adequate in number and present in thin new bone trabeculae (Figure 10). Osteoblasts were seen as a rim of cells on the surface of the bone, a new bone trabecula lined by osteoblast cells, and active osteoid tissue. There was proof that the newly formed bone had matured to form primary osteons. The space between the implant surfaces and the parent bone bed was bridged by the immature woven bone (Figure 10).

**Figure 11:**
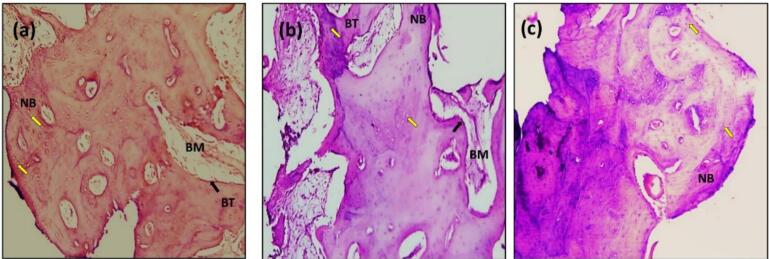
Microscopic photograph view of a cross-section of the Propolis laser implant treatment: (a) 14 days; (b) 90 days; (c) 180 days. (NB): new bone, (BT): bone trabeculae, (BM): bone marrow. Black arrow: Osteoblasts (Ob); Yellow arrow: Osteocytes (Oc); (Hematoxylin-Eosin, magnification = 10X)

 After 14 days of implantation, the histologic features of the implants coated with propolis revealed the formation of new osteon tissue, numerous bone cells nearby, and capillaries, as demonstrated in Figure 11. Many osteoblast cells line a newly formed bone trabecula, and osteoblasts were grouped as a rim of cells on the surface of the bone and active osteoid tissue. The threads in the marrow healing space following the thread of the implant showed bone trabeculae, an adequate number of osteocytes within lacunae embedded in bone trabeculae, raw osteoblasts arranged on the periphery of the trabeculae with a sufficient amount of bone matrix around the implants.

 In the 90-day healing period, implants appeared to be embedded in the bone, with no signs of inflammation within the peri-implant soft tissues.

 The sections with H&E staining showed new bone formation (NBF) with increased osteocyte cells. The slides demonstrated the active process of bone formation as evidenced by the large number of active osteocytes irregularly scattered in thick trabeculae, with a reduced number of osteoblasts and the absence of osteoclasts (Figures 8, 9, 10, and 11b). The light microscopic analysis showed differences in the bone response to the different surface treatments of the implant groups.

 At 90 days, bone ingrowth had increased, and the new bone had matured in all the implant groups, especially in sandblasting and propolis-treated groups (Figures 9 and 11b) compared with 14 days of implantation; compact lamellar bone filled most of the gap area. There was also active remodeling with a mixed pattern of densely packed mature and immature bone, deep pink new bone detected, and bone ingrowth that had advanced close to all implant surfaces. The bone-to-implant interface showed no signs of fibrous connective tissue, and the distinction between new and old bone was blurred.

 After 180 days, the sections with H&E staining and the light microscopic analysis showed fewer differences between implant-treated groups (A, B, C, and D). Without an intervening fibrous tissue layer, the bone along the implant surface had been remodeled, and osteoid was deposited (Figures 8, 9, 10, 11c). The sections exhibiting basic staining demonstrated a remarkable remodeling of the bone surrounding the implant, with all implants encircled by dense bone apposition close to the implant and in direct contact with the more mature bone. After 180 days, the development of osteons along the implant’s surface and the replacement of woven bone with lamellar bone were indicators of the maturation of the bone surrounding the implants.

 Ongoing osteoconductive bone formation towards the grooves could be observed in more detail. The thick bone trabeculae were in contact with the delayed-loaded implant surfaces. A characteristic demarcation between old and new bone was apparent. Numerous osteocytes were seen in their lacunae in the mature woven (lamellar) bone.

###  New bone formation at different time intervals

 The results of NBF showed that the bone percentage mean values for all the implant groups had increased over time from 14 to 90 to 180 days (Table 1). One-way ANOVA of the NBF with different implant surface treatments (Table 2) showed a significant difference in the NBF at different time intervals.

**Table 1:** Means and standard deviations of the NBF (µm) at different time intervals

**Time (day)**	**Surface treatments, Mean±SD**				**N**
	**Sandblasting plus acid etching**	**Sandblasting**	**Laser treatment**	**Propolis coating**	
14	9.33 ± 2.36d	9.16 ± 1.44 ^e^	8.50 ± 1.32f	9.16 ± 2.36 ^e^	4
90	10.66 ± 0.57 ^c^	15.00 ± 4.35a	7.16 ± 2.46h	11.66 ± 0.76 ^b^	4
180	10.66 ± 2.08 ^c^	11.00 ± 1.73 ^c^	8.16 ± 2.46g	11.66 ± 1.52b	4

SD, Standard deviation, number of slide sections = 4. Different letters are significantly different according to Tukey test.

**Table 2:** One-way ANOVA of the NBF with different implant surface treatments

	**Sum of squares**	**df**	**Mean square**	**F- value**	***P*** ** value**
Between groups	142.576	11	12.961	2.741	0.019*
Within groups	113.500	24	4.729		
Total	256.076	35			

*Significant differences; df, degree of freedom.

 In the 14-day healing period, the results of the implant surface treatment showed that the sandblasting plus acid etching group had the highest mean value of NBF (9.33 µm) than the sandblasting and propolis coating groups, while the laser surface treatment group had the lowest mean value (8.50 µm) concerning NBF (Table 1).

 In the 90 days healing period, the results of the implant surface treatment showed that the sandblasting group had the highest mean value (15 µm) for NBF than the propolis coating and sandblasting plus acid etching groups, while the laser surface treatment group had the lowest mean value (7.16 µm) (Table 1). In the 180 days healing period, the results of the implant surface treatment showed that the propolis coating group had the highest mean value (11.66 µm) for NBF than the sandblasting and sandblasting plus acid etching groups, while the laser surface treatment group had the lowest mean value (8.16 µm) (Table 1).

###  Bone cell count at different time intervals

 The bone cell count, osteoblast (OB), osteocyte (Oc), and osteoclast (OC) cells associated with each treated implant group (groups A, B, C, and C) were examined and compared for their count at 14, 90, and 180 days as shown in Table 3. One-way ANOVA (Table 4) showed a significant difference in the mean value of osteoblasts and osteocytes for sandblasting with acid etching, sandblasting, laser, and propolis surface treatments (*P* ˂ 0.05) at a 14-, 90-, and 180-day interval. However, there were no significant differences at different time intervals for osteoclast cells.

**Table 3:** Mean and standard deviation of the bone cell count with different time intervals

**Bone cell count, Mean±SD**						
**Cell type**	**Time (day)**	**Sandblast +acid etching**	**Sandblasting**	**Laser treatment**	**Propolis**	***P *** **value**
OB	14	15 ± 9.97^b^	10 ± 5.56 ^c^	9 ± 4.69^c^	17 ± 8.7^a^	0.000
	90	13 ± 1.50^b^	9 ± 6.05 ^c^	3 ± 2.94^d^	1 ± 1.25^e^	
	180	16 ± 7.85^a^	1 ± 1.29 ^e^	1 ± 1.91^e^	3 ± 1.70^d^	
Oc	14	14_ _± 9.30^g^	21 ± 10.66d	17 ± 9.57^f^	18 ± 9.5^f^	0.059
	90	21 ± 8.53^d^	23 ± 2.50 ^c^	19 ± 5.19^h^	26 ± 4.2^b^	
	180	27 ± 11.12^a^	25 ± 7.16 ^e^	26 ± 4.78^b^	29 ± 2.9^c^	
OC	14	1 ± 0.00	1 ± 0.001	1 ± 0.00	1 ± 0.00	0.913
	90	1 ± 0.00	1 ± 0.00	0 ± 0.00	1 ± 0.00	
	180	0 ± 0.00	0 ± 0.00	0 ± 0.00	0 ± 0.00	

SD, Standard deviation; OB, osteoblast; OC, osteocyte; OC, osteoclast. Different letters are significantly different according to Tukey tests.

**Table 4:** One-way ANOVA of the bone cell count with different implant surface treatments

		**Sum of squares**	**df**	**Mean square**	**F-value**	***P*** ** value**
OB	Between groups	1710.417	11	155.492 28.986	5.364	0.000*
	Within groups	1043.500	36			
	Total	2753.917	47			
Oc	Between groups	1156.917	11	105.174 59.583	1.765	0.059*
	Within groups	2145.000	36			
	Total	3301.917	47			
OC	Between groups	0.972	11	0.088 0.194	0.455	0.913
	Within groups	4.667	24			
	Total	5.639	35			

*Significant differences; df, degree of freedom.

 In Table 3, the results of the osteoblasts showed a significant reduction at 90 days for all implant-tested groups, with a significant increase in osteocytes after 90 and 180 days compared to 14 days for all implant-treated groups. Osteoclast cell counts did not significantly decrease after 90 and 180 days compared to 14 days in all the implant groups.

Table 3 shows the comparison of cell type counts after 14, 90, and 180 days. The highest osteoblast cell mean value (17) was recorded in the propolis group at the 14-day interval. At 90- and 180-day intervals, the highest osteoblast cell mean value (16) was recorded in the sandblast plus acid etch group. The highest osteocyte mean value (21) was recorded in the sandblasting group at the 14-day interval. At 90- and 180-day intervals, the highest osteocyte cell mean value (29) was recorded in the propoliscoating implant group at the 180-day interval.

## Discussion

 The current study evaluated dental implant osseointegration in dog models by assessing the effects of sandblasting, sandblasting combined with acid etching, Er,Cr:YSGG laser, and propolis coating of implant surfaces under occlusal stress.

 In the present study, radiographic (Figure 6) and histologic findings (Figures 8, 9, 10, and 11) revealed a higher rate of osseointegration and bone density at peri-implant tissue in the loaded implant after a 180-day follow-up period compared to the unloaded implant 14 and 90 days after implant installation.

 The variations in surface energy due to implant sandblasting with Al_2_O_3_ or combined with acid etching alter the implant surface’s hydrophilicity, wettability, and possibility for early interaction with biological fluids (changes in the chemistry of the surface).The increased surface area of dental implants with rough surface characteristics should enhance bone ingrowth.^15^

 The implant stability is improved by surface changes that increase titanium roughness (beneficial for osteoblast proliferation and bone formation).The surface topography of dental implants is crucial for osteoblast adhesion and differentiation during the early phases of osseointegration and long-term bone remodeling.^16^

 It has been demonstrated that sandblasting methods produce a negative surface charge. According to Guo et al,^17^ a negative surface charge enhances cell adhesion and osteoblastic development, making it easier for protein adsorption, which is needed for cellular growth and higher bone production.^18,19^

 Hsu et al reported that surface irregularities allow osteogenic cells to bind and deposit bone, improving the mechanical interlocking between the macromolecules of the implant surface and the bone and creating bone-to-implant contact.^20^

 As a result, this study focused on employing an Er,Cr:YSGG laser to modify the titanium implant surface (Figures 7c and 10). This laser can produce complex surface geometries and biomedical implant surfaces since it can quickly produce high-resolution complex microstructures with free contamination at the nano- and micrometer scales. In other words, we can claim that laser irradiation considerably altered the rough surface of this implant.^21^

 Based on the findings (Figures 7d and 11), propolis coating has also been used in dental implants to speed up osseointegration. Plant resins, which are responsible for various biological activities, are the source of flavonoids, which are thought to be an essential biochemical component in propolis. Numerous studies have examined the effects of flavonoids in cell culture and animal model systems, and the results of these investigations have supported evidence for the role of flavonoids in both in vitro and in vivo bone formation.

 The functional properties of propolis components analyzed in the current study can explain the clinical impact of these components on the osseointegration of the titanium implant. Research has explained the effect of propolis flavonoids and shown potential antimicrobial activity through its biofilm inhibitory capacity of pathogens (*Escherichia coli* and *Staphylococcus aureus*).^22^

 Most flavonoids exert an effect on bone by promoting osteoblast genesis, which ultimately leads to bone formation. Although no research studies on propolis-coated dental implants on canine models are ongoing, there is still some uncertainty regarding the long-term stability of these coatings, and they are currently employed clinically on a small percentage of clinical implants.^23,24^

 Numerous investigations have employed different animal models to assess the impacts of loading and overloading on peri-implant tissues. The expected results are affected by the anatomical conditions, the experimental technique, the implant design, and the presence or absence of plaque control.

 The current study appears to have only considered the centric occlusion, leaving molars in the mandible to support the occlusion. The screw-retained prosthesis supported by implants installed in the mandible was carefully placed in contact with the upper jaw’s natural teeth, and an excessive load on the implants supporting the prosthesis could not be excluded.

 Moreover, dogs use their strong, long tooth crowns to chew and apply an overload when transmitting food in the molar region. They do this using physiologic chewing for both centric and lateral occlusion instead of the four movements performed by humans.^25,26^ As a result, the load is distributed to the natural residual teeth and implants placed in healed sites supporting fixed prostheses.

 A load applied to the chewing elements in humans does not appear to be likely reproduced correctly in dogs. Most research up to this point has concentrated on the occlusal contacts of flat anatomical surfaces in centric occlusion. In the current study, metal crowns have been applied to the loaded areas with prosthetic restorations with anatomical surfaces.

 However, the dental/implant occlusion is more complex than just a simple surface-to-surface contact. It is complicated and involves many force vectors (axial and nonaxial) and the friction of several inclined planes. These force vectors are transient and occur simultaneously. Consequently, the concept of multidirectional occlusal forces should be used in place instead of a unidirectional occlusal force.^27^

 Bone healing causes an increase in the amount of contact between the implant and the surrounding bone after insertion. An implant may fail if loaded before this contact reaches a particular point. Because of this, the implant stability result should be used to determine and decide the loading time. Undermining load may impact the rate of implant survival and osseointegration or bone density surrounding implants, according to several studies on the premature contacts incorporated into the prosthesis.^28^ The loaded implant creates a dynamic complex where forces, materials, interfaces, cells, and bone tissue interact in a planned way to accomplish and sustain osseointegration, implant stability, and healthy bone function over time.^29^

 To ensure proper communication mechanisms among the participating cells, the bone cells (osteoblasts, osteocytes, and osteoclasts) participate in the remodeling process in various skeletal areas asynchronously, directed by local and regulated general factors.^30^ In response to mechanical loads and other stimuli, the balance is maintained between osteoblasts, which synthesize new bone tissue, and osteoclasts, which dissolve the bone matrix. Osteocytes regulate the activity of both osteoblasts and osteoclasts.^31^ The limitations of the current in vivo study concern extrapolating human results from this animal study. Canines can only perform two movements, a vertical and a lateral. In contrast, people can perform four motions, with significant variations in tooth form and deglutition, making it impossible for canines to replicate human occlusion and function. Further research is needed due to the lack of survival rate or long-term success of titanium implants.

## Conclusion

 A radiographic examination revealed that surface modification had a significant impact on osseointegration, with a strong bond between the implant surface and the surrounding bone in the apical portion of the implant. The histological sections at the 14-day interval revealed obvious bone remodeling activities, especially in the sandblasting plus acid etching and the sandblasting groups with a uniform and continuous pattern compared with the other implant-modified groups. At the 90-day interval, bone ingrowth had increased, and the new bone had matured in all the implant groups, especially in the sandblasting and propolis surface modification groups. After 180 days, the delayed-loaded implant groups’ histological sections showed differences between different implant groups with a remarkable remodeling of the bone surrounding the implants, especially in the propolis coating group.

## Acknowledgments

 The authors express their gratitude to the College of Dentistry and the College of Veterinary Medicine, University of Mosul for their assistance, availability of the laboratory, and contributions that facilitated the study.

## Competing Interests

 There were no conflicts of interest regarding this research.

## Ethical Approval

 The present study obtained ethics approval from the Research Ethics Committee, College of Dentistry, University of Mosul, Mosul, Iraq (REC reference: UoM. Dent/A.67/22). This experimental animal study was conducted at the experimental surgical center of the Veterinary Teaching Hospital, the College of Veterinary Medicine, University of Mosul.

## Funding

 Self-funded.

## References

[1] Elias CN, Fernandes DJ, de Souza FM, dos Santos Monteiro E, de Biasi RS (2019). Mechanical and clinical properties of titanium and titanium-based alloys (Ti G2, Ti G4 cold worked nanostructured and Ti G5) for biomedical applications. J Mater Res Technol.

[2] Bruns S, Krüger D, Galli S, Wieland DC, Hammel JU, Beckmann F (2023). On the material dependency of peri-implant morphology and stability in healing bone. Bioact Mater.

[3] Patel V, Sadiq MS, Najeeb S, Khurshid Z, Zafar MS, Heboyan A (2023). Effects of metformin on the bioactivity and osseointegration of dental implants: a systematic review. J Taibah Univ Med Sci.

[4] Huang YC, Huang YC, Ding SJ (2023). Primary stability of implant placement and loading related to dental implant materials and designs: a literature review. J Dent Sci.

[5] Schünemann FH, Galárraga-Vinueza ME, Magini R, Fredel M, Silva F, Souza JC (2019). Zirconia surface modifications for implant dentistry. Mater Sci Eng C Mater Biol Appl.

[6] Kim HK, Ahn B (2021). Effect of Al2O3 sandblasting particle size on the surface topography and residual compressive stresses of three different dental zirconia grades. Materials (Basel).

[7] Jambhulkar N, Jaju S, Raut A, Bhoneja B (2023). A review on surface modification of dental implants among various implant materials. Mater Today Proc.

[8] Studenikin RV, Mamedov AA. Stability of short and long dental implants placed at different levels. Medical Alphabet. 2022(2):17-24. 10.33667/2078-5631-2022-2-17-24.

[9] Sargolzaie N, Rafiee M, Salari Sedigh H, Zare Mahmoudabadi R, Keshavarz H (2018). Comparison of the effect of hemihydrate calcium sulfate granules and Cerabone on dental socket preservation: an animal experiment. J Dent Res Dent Clin Dent Prospects.

[10] Velasco-Ortega E, Matos-Garrido N, Jiménez-Guerra A, Ortiz-Garcia I, Moreno-Muñoz J, Núñez-Márquez E (2023). Early loading of two implants supporting mandibular overdentures in geriatric edentulous patients: a 12-year follow-up study. J Clin Med.

[11] Vergara-Buenaventura A, Villanueva M, Montoya JP, Mendoza-Azpur G (2019). A literature review on progressive loading. J Osseointegration.

[12] Kung PC, Hsu CW, Yang AC, Chen NY, Tsou NT (2023). Prediction of bone healing around dental implants in various boundary conditions by deep learning network. Int J Mol Sci.

[13] Bellows J. Small Animal Dental Equipment, Materials, and Techniques. Hoboken, NJ: John Wiley & Sons; 2019. 10.1002/9781118986646.

[14] Abdulla MA, Hasan RH, Al-Hyani OH (2023). Impact of Er,Cr:YSGG laser, sandblast, and acid etching surface modification on surface topography of biodental titanium implants. J Lasers Med Sci.

[15] Moslehifard E, Seyyedashrafi MM, Khosronejad N (2021). Evaluation of surface roughness of a Ni-Cr alloy treated with the Nd/YAG laser and the sandblast technique. J Lasers Med Sci.

[16] Matos GR (2021). Surface roughness of dental implant and osseointegration. J Maxillofac Oral Surg.

[17] Guo CY, Matinlinna JP, Tsoi JK, Hong Tang AT (2019). Residual contaminations of silicon-based glass, alumina and aluminum grits on a titanium surface after sandblasting. Silicon.

[18] de Jesus RN, Carrilho E, Antunes PV, Ramalho A, Moura CCG, Stavropoulos A (2018). Interfacial biomechanical properties of a dual acid-etched versus a chemically modified hydrophilic dual acid-etched implant surface: an experimental study in Beagles. Int J Implant Dent.

[19] Cervino G, Fiorillo L, Iannello G, Santonocito D, Risitano G, Cicciù M (2019). Sandblasted and acid etched titanium dental implant surfaces systematic review and confocal microscopy evaluation. Materials (Basel).

[20] Hsu SH, Liu BS, Lin WH, Chiang HC, Huang SC, Cheng SS (2007). Characterization and biocompatibility of a titanium dental implant with a laser irradiated and dual-acid etched surface. Biomed Mater Eng.

[21] Gholami GA, Karamlou M, Fekrazad R, Ghanavati F, Hakimiha N, Romanos G (2018). Comparison of the effects of Er,Cr:YSGG laser and super-saturated citric acid on the debridement of contaminated implant surfaces. J Lasers Med Sci.

[22] Al-Shabib NA, Husain FM, Ahmad I, Khan MS, Khan RA, Khan JM (2017). Rutin inhibits mono and multi-species biofilm formation by foodborne drug resistant Escherichia coli and Staphylococcus aureus. Food Control.

[23] Ramesh P, Jagadeesan R, Sekaran S, Dhanasekaran A, Vimalraj S (2021). Flavonoids: classification, function, and molecular mechanisms involved in bone remodelling. Front Endocrinol (Lausanne).

[24] Woźniak M, Sip A, Mrówczyńska L, Broniarczyk J, Waśkiewicz A, Ratajczak I (2022). Biological activity and chemical composition of propolis from various regions of Poland. Molecules.

[25] Passerini A, Vigano P, Cesaretti G, Rodríguez Sosa VM, Cabrera García A, Soca Pérez M (2017). New point of view of masticatory dynamic in dog. Rev Electron Vet.

[26] Cesaretti G, Lang NP, Viganò P, Bengazi F, Apaza Alccayhuaman KA, Botticelli D (2018). Immediate and delayed loading of fixed dental prostheses supported by single or two splinted implants: a histomorphometric study in dogs. J Oral Rehabil.

[27] Katona TR, Eckert GJ (2017). The mechanics of dental occlusion and disclusion. Clin Biomech (Bristol, Avon).

[28] Allahbakhshi H, Vafaee F, Lotfazar M, Ahangary AH, Khoshhal M, Fotovat F (2017). Immediate vs delayed endosseous integration of maxi implants: a torque removal animal study. J Dent Res Dent Clin Dent Prospects.

[29] Lin TH, Tamaki Y, Pajarinen J, Waters HA, Woo DK, Yao Z (2014). Chronic inflammation in biomaterial-induced periprosthetic osteolysis: NF-κB as a therapeutic target. Acta Biomater.

[30] Corrigan MA, Johnson GP, Stavenschi E, Riffault M, Labour MN, Hoey DA (2018). TRPV4-mediates oscillatory fluid shear mechanotransduction in mesenchymal stem cells in part via the primary cilium. Sci Rep.

[31] Sims NA, Martin TJ (2014). Coupling the activities of bone formation and resorption: a multitude of signals within the basic multicellular unit. Bonekey Rep.

